# Supplementary material for: The adverse effects of trastuzumab-containing regimes as a therapy in breast cancer: A piggy-back systematic review and meta-analysis
Source: PLoS One. 2022 Dec 1;17(12):e0275321. doi: 10.1371/journal.pone.0275321 (PMC9714930; doi:10.1371/journal.pone.0275321)
Supplement: S1 File — (DOCX) [file pone.0275321.s002.docx]

# Supporting Information 1

Characteristics of the trials identified in search

RCTs comparing trastuzumab in one arm versus no trastuzumab in another (used in meta-analysis)

| **Trial name(s)** | **Key references** | **Drug regimes in each arm** | **Chemo regimes** | **Other HER2 drugs** | **Included/excluded?** |
| --- | --- | --- | --- | --- | --- |
| **Trials with matched arms of trastuzumab v no trastuzumab** | | | | | |
| HERA  BIG 1-01  NCT00045032 | [1–4] | 1. 24 months trastuzumab  2. 12 months trastuzumab  3. no trastuzumab  Mixed chemotherapies prior to trastuzumab  (radiotherapy & hormone therapy allowed) | Trial started after chemotherapy | Trastuzumab  v nothing | Included |
| NSABP B31  NCT00004067 | [5,6] | 1. trastuzumab & doxorubicin & cyclophosphamide & paclitaxel (ACP)  2. doxorubicin & cyclophosphamide & paclitaxel (ACP) | Taxanes & AC | Trastuzumab  v nothing | Included |
| NCCTG N9831  NCT00005970 | [7,8] | 1. doxorubicin & cyclophosphamide & paclitaxel (ACP) & then trastuzumab  2. doxorubicin & cyclophosphamide & paclitaxel (ACP) & concurrent trastuzumab  3. doxorubicin & cyclophosphamide & paclitaxel (ACP)  4. doxorubicin & cyclophosphamide & paclitaxel (ACP) – Arm 1 before trast | Taxanes & AC | Trastuzumab  v nothing  *(using arms 2v3)* | Included |
| BCIRG 006,  UCLA-0102006  NCT00021255 | [9,10] | 1. doxorubicin & cyclophosphamide (AC) & trastuzumab  2. docetaxel (carboplatin) & trastuzumab  3. doxorubicin & cyclophosphamide (AC) then docetaxel | AC  Taxanes & carboplatin | Trastuzumab  v nothing  *(using arms 1v3)* | Included |
| FNCLCC PACS-04  NCT00054587 | [11] | 1. FEC then 12 months trastuzumab  2. epirubicin & docetaxel (ED) then 12 month trastuzumab  3. FEC  4. ED  Radiotherapy & hormone therapy allowed | FEC  v Taxanes & epirubicin | Trastuzumab  v nothing  *(using arms 1v3 and 2v4 separately)* | Included |
| NOAH | [12] | 1. neoadjuvant chemotherapy (FEC & doxorubicin & paclitaxel) & 12 months trastuzumab  2. neoadjuvant chemotherapy only (HER2+)  3. neoadjuvant chemotherapy only (HER2-)  (Radiotherapy & hormone therapy allowed) | FEC & taxanes | Trastuzumab  v nothing  *(using arms 1v2)* | Included |
| eLEcTRA  NCT00171847 | [13] | 1. Trastuzumab  2. None  3. None (HER2-)  (All patients had letrozole hormone therapy)  Previous trastuzumab was allowed but no proportion given | None | Trastuzumab  v nothing  *(using arms 1v2)* | Included |
| Gasparini | [14] | 1. paclitaxel & trastuzumab  2. paclitaxel | Taxane | Trastuzumab  v nothing | Included |
| H0649g - 1 | [15] | 1. Doxorubicin/epirubicin & cyclophosphamide (AC) & trastuzumab  2. paclitaxel & trastuzumab  3. AC  4. paclitaxel  Chemotherapy groups not randomised – depended on prior treatment | AC  v taxanes | Trastuzumab  v nothing  *(using arms 1v3)* | Included  (adverse effects reported for arms 1&3) |
| MD Anderson 2005 | [16] | 1. paclitaxel & FEC  2. paclitaxel & FEC & 6 months trastuzumab  (all had radiotherapy, hormone therapy) | FEC & taxanes | Trastuzumab  v nothing | Included |
| H2269s  NCT00068341 | [17,18] | 1. neoadjuvant/adjuvant docetaxel & carboplatin (TC) & neoadjuvant trastuzumab and 12 months adjuvant trastuzumab  2. neoadjuvant/adjuvant docetaxel & carboplatin (TC) and 12 months adjuvant trastuzumab  3. neoadjuvant/adjuvant docetaxel & carboplatin (TC)  N.B. Patients in Arm 3 HER2-  (other arm of HER2+ received adjuvant trastuzumab) | Taxanes & carboplatin | Trastuzumab  v nothing  *(using arms 2v3)* | Included |
| CALGB 9840  NCT00003440 | [19] | 1. Paclitaxel & trastuzumab  2. Paclitaxel  (HER2- only) | Taxanes | Trastuzumab  v nothing | Included  (severe adverse events only reported) |
| ABCSG-24  NCT00309556 | [20] | 1. Epirubicin & docetaxel & capecitabine & trastuzumab  2. Epirubicin & docetaxel & capecitabine  3. Epirubicin & docetaxel & trastuzumab  4. Epirubicin & docetaxel | AC & taxanes  v  Taxanes & epirubicin | Trastuzumab  v nothing | Included  (adverse events not reported separately for different chemo regimes) |
| REMAGUS 02 | [21] | 1. Docetaxel & cyclophosphamide & epirubicin (neoadjuvant)  2. Docetaxel & cyclophosphamide & epirubicin & trastuzumab (neoadjuvant)  (patients received adjuvant therapy too, but side effects noted after AC and then after taxane +/- trastuzumab) | AC & taxanes | Trastuzumab  v nothing | Included |
| TAnDEM trial  NCT00022672 | [22,23] | 1. Trastuzumab  2. None  Previous chemotherapy allowed more than 6 months prior, no prior trastuzumab  (all also had anastrozole hormone therapy) | None  (hormone only) | Trastuzumab  v nothing | Included |
| M77001 | [24] | 1. Trastuzumab & docetaxel  2. Docetaxel | Taxanes | Trastuzumab  v nothing | Included |
| GeparQuinto  GBG 44  (run-in phase only)  NCT00567554 | [25] | (HER2+ arms only)  1. Epirubicin & cyclophosphamide & docetaxel & trastuzumab  2. docetaxel & trastuzumab  (HER2- arm)  3. Epirubicin & cyclophosphamide & docetaxel  4. docetaxel  Adverse effects reported for chemotherapy alone and in combination with HER2 agent in Table 1, although not clear how that relates to trial protocol in Fig 1 of paper. | EC  v docetaxel | Trastuzumab  v nothing | Included  (adverse effects reported for different subsets in run-in phase) |
| FinHer | [26] | 1. FEC & docetaxel & trastuzumab  2. FEC & vinorelbine & trastuzumab  3. FEC & docetaxel  4. FEC & vinorelbine | FEC & taxanes  v  FEC & vinorelbine | Trastuzumab  v nothing | Included |
| GBG 26  BIG 3-05  NCT00148876 | [27–29] | 1. capecitabine & trastuzumab  2. capecitabine  All patients had had previous treatment, including with trastuzumab | Capecitabine | Trastuzumab  v nothing | Included |
| NSABP B-47  NCT01275677 | [30,31] | 1. docetaxel & cyclophosphamide  2. paclitaxel, doxorubin & cyclophosphamide  3. docetaxel & cyclophosphamide & 12 months trastuzumab  4. paclitaxel, doxorubin & cyclophosphamide & 12 months trastuzumab  Some patients given hormone therapy or radiotherapy as clinically indicated  Adverse events reported only for paired arms 1& 2 (no trastuzumab) and 3 & 4 (trastuzumab) together | Taxanes & anthracyclines (clinician choice) | Trastuzumab  v nothing | Included |
| NSABP B-43  NCT00769379 | [32,33] | 1. No trastuzumab 2. Trastuzumab   All women had radiotherapy. Those for whom it was suitable had hormone therapy (balanced across arms) | None | Trastuzumab  v nothing | Included |
| NCT00971737 | [34] | 1. Cyclophosphamide & GM-CSF-secreting breast cancer vaccine 2. Cyclophosphamide & GM-CSF-secreting breast cancer vaccine & trastuzumab | Cyclophosphamide | Trastuzumab  v nothing | Included |

| **Trials containing trastuzumab v lapatinib** | | | | | |
| --- | --- | --- | --- | --- | --- |
| TRIO-US B07  NCT00769470 | [35] | 1. Docetaxel & carboplatin & trastuzumab  2. Docetaxel & carboplatin & lapatinib  3. Docetaxel & carboplatin & trastuzumab & lapatinib | Taxanes & carboplatin | Trastuzumab  v lapatinib  v trastuzumab & lapatinib | Data extracted but not included in published analyses |
| ALTTO  BIG 2-06  NCCTG  N063D  NCT00490139 | [36] | 1. trastuzumab  2. lapatinib & trastuzumab  3. trastuzumab THEN lapatinib  4. lapatinib  Some also received chemotherapy  (radiotherapy & hormone therapy allowed) | Mixed | Trastuzumab  v trastuzumab  & lapatinib  v lapatinib | Data extracted but not included in published analyses |
| GEICAM/2006-14  2007-007031-13  NCT00841828 | [37] | 1. Epirubicin & cyclophosphamide (AC) then docetaxel & lapatinib  2. Epirubicin & cyclophosphamide (AC) then docetaxel & trastuzumab  Any other treatments allowed | AC & taxanes | Trastuzumab  v lapatinib | Data extracted but not included in published analyses |
| EORTC 10054  NCT00450892 | [38] | 1. Docetaxel then FEC & lapatinib  2. Docetaxel then FEC & trastuzumab  3. Docetaxel then FEC & trastuzumab & lapatinib | FEC & taxanes | Trastuzumab  v lapatinib  v trastuzumab & lapatinib | Data extracted but not included in published analyses |
| CALGB 40601  NCT00770809 | [39,40] | 1. Trastuzumab & paclitaxel & lapatinib  2. Trastuzumab & paclitaxel  3. Paclitaxel & lapatinib (closed early) | Taxanes | Trastuzumab  v lapatinib  v trastuzumab & lapatinib | Data extracted but not included in published analyses |
| NCIC CTG MA.31  EGF108919  NCT00667251 | [41,42] | 1. Paclitaxel or docetaxel & lapatinib  2. Paclitaxel or docetaxel & trastuzumab  All prior treatments allowed but trastuzumab or taxane had to be >12 months previously. 18% had prior trastuzumab therapy | Taxanes | Trastuzumab  v lapatinib | Data extracted but not included in published analyses  Adverse events recorded during combined and monotherapy separately |
| CEREBEL  EGF111438  NCT00820222 | [43,44] | 1. Capecitabine & trastuzumab  2. Capecitabine & lapatinib  Patients had mostly had prior chemotherapy and about half had had prior trastuzumab therapy | Capecitabine | Trastuzumab  v lapatinib | Data extracted but not included in published analyses |
| CHER-LOB  NCT00429299 | [45,46] | 1. paclitaxel then FEC & trastuzumab  2. paclitaxel then FEC & lapatinib  paclitaxel then FEC & trastuzumab & lapatinib  (no other treatments allowed) | Taxanes & FEC | Trastuzumab  v lapatinib  v trastuzumab & lapatinib | Data extracted but not included in published analyses |
| NSABP B-41  NCT00486668 | [47] | 1. Doxorubicin & cyclophosphamide (AC) then paclitaxel & trastuzumab (neoadjuvant) with adjuvant trastuzumab  2. AC then paclitaxel & lapatinib (neoadjuvant) with adjuvant trastuzumab  3. AC then paclitaxel & trastuzumab & lapatinib (neoadjuvant) with adjuvant trastuzumab | AC & taxanes | Trastuzumab  v lapatinib  v trastuzumab & lapatinib | Data extracted but not included in published analyses |
| NeoALTTO  BIG 01−06  EGF 106903  NCT00553358 | [48–50] | 1: lapatinib & paclitaxel  2: trastuzumab & paclitaxel  3: lapatinib & trastuzumab & paclitaxel  All neoadjuvant, followed by FEC | FEC & taxanes | Trastuzumab  v lapatinib  v trastuzumab & lapatinib | Data extracted but not included in published analyses |
| ALTERNATIVE  NCT01160211 | [51,52] | 1. trastuzumab & lapatinib  2. lapatinib  3. trastuzumab  Aromatase inhibitor hormone therapy  All patients had previously been treated with chemotherapy & trastuzumab – 1/3^rd^ in adjuvant setting (2/3^rd^ neoadjuvant) | Mixed | Trastuzumab  v lapatinib  v trastuzumab & lapatinib | Data extracted but not included in published analyses |
| NCT00524303  LPT109096 | [53,54] | 1. FEC & paclitaxel & trastuzumab  2. FEC & paclitaxel & lapatinib  3. FEC & paclitaxel & trastuzumab & lapatinib  No concurrent therapies | FEC & taxanes | Trastuzumab  v lapatinib  v trastuzumab & lapatinib | Data extracted but not included in published analyses |
| EGF104900  NCT00320385 | [55,56] | 1. Lapatinib  2. Lapatinib & trastuzumab  (all previous treated with trastuzumab) |  | Trastuzumab  v trastuzumab & lapatinib | Data extracted but not included in published analyses |
| ELTOP  WJOG 6110B | [57] | 1. Capecitabine, lapatinib & trastuzumab 2. Capecitabine, lapatinib   (all previous treated with taxanes and trastuzumab) | Capecitabine | Trastuzumab & lapatinib  v lapatinib | Not in published analyses |

RCTs comparing trastuzumab emtansine with trastuzumab or chemotherapy alone

| **Trial name(s)** | **Key reference** | **Drug regimes in each arm** | **Chemo regimes** | **Other HER2 drugs** | **Included/excluded?** |
| --- | --- | --- | --- | --- | --- |
| ADAPT HER2+/HR+ | [58,59] | 1. Trastuzumab emtansine  2. Trastuzumab  (hormone therapy allowed) | T-DM1 v trastuzumab |  | Data extracted but not included in published analyses |
| KRISTINE  NCT02131064 | [60] | 1. Pertuzumab & trastuzumab & docetaxel & carboplatin  2. Pertuzumab & trastuzumab emtansine | Taxanes & carboplatin  v  T-DM1 | Pertuzumab & trastuzumab  v  Pertuzumab & T-DM1 | Data extracted but not included in published analyses |
| TDM4450g  NCT00679341 | [61,62] | 1. Trastuzumab emtansine  2. Docetaxel & trastuzumab  25% had had prior trastuzumab therapy; almost all had had prior chemotherapy more than 6 months before | Taxanes  v  T-DM1 |  | Data extracted but not included in published analyses |
| MARIANNE  NCT01120184 | [63] | 1. Docetaxel/paclitaxel & trastuzumab  2. Trastuzumab emtansine & placebo  3. Trastuzumab emtansine & pertuzumab | Taxanes  T-DM1 | Trastuzumab  v T-DM1  v T-DM1 & pertuzumab | Data extracted but not included in published analyses |
| KATHERINE  NCT01772472 | [64] | 1. Trastuzumab  2. Trastuzumab emtansine  All patients had previously received trastuzumab (and taxane chemotherapy) |  | T-DM1  v trastuzumab | Data extracted but not included in published analyses |
| EMILIA  NCT00829166 | [65] | 1. Trastuzumab emtansine  2. Capecitabine & lapatinib  All had previously had trastuzumab and taxane chemotherapy | Capecitabine  T-DM1 | T-DM1  v lapatinib | Data extracted but not included in published analyses |
| TDM4652g  NCT00951665 | [66,67] | 1. Paclitaxel & trastuzumab emtansine  2. Paclitaxel & trastuzumab emtansine & pertuzumab  Patients had mostly already received taxanes (82%) and trastuzumab (985) | Taxanes | T-DM1  v  T-DM1 & pertuzumab | Data extracted but not included in published analyses |
| ATEMPT  NCT01853748 | [68] | 1. Trastuzumab Emtansine 2. Paclitaxel & trastuzumab |  | T-DM1  v trastuzumab | Not included in published analyses |
| PREDIX HER2 | [69] | 1. Docetaxel, trastuzumab & pertuzumab 2. Trastuzumab emtansine |  | Trastuzumab & pertuzumab  v  T-DM1 | Not included in published analyses |
| Neopeaks  JBCRG association study-20 | [70] | 1. Docetaxel, carboplatin, trastuzumab & pertuzumab 2. Docetaxel, carboplatin, trastuzumab & pertuzumab followed by trastuzumab emtansine 3. Trastuzumab emtansine and pertuzumab (personalised treatment depending on response) |  |  | Not included in published analyses |
| NCT01966471 | [71] | 1. Trastuzumab, pertuzumab & taxane 2. Trastuzumab Emtansine and pertuzumab   All patients also had anthracycline chemotherapy first | Taxane  v  T-DM1 |  | Not included in published analyses |
| NCT02144012 | [72] | 1. Trastuzumab emtansine 2. Trastuzumab & docetaxel | Taxane  v  T-DM1 |  | Not included in published analyses  (terminated early) |

RCTs comparing trastuzumab in both arms but with different chemotherapy (eg taxanes v anthracyclines)

| **Trial name(s)** | **Key reference** | **Drug regimes in each arm** | **Chemo regimes** | **Other HER2 drugs** | **Included/excluded?** |
| --- | --- | --- | --- | --- | --- |
| ADAPT HER2+/HR- | [58,59] | 1. Trastuzumab & pertuzumab  2. Trastuzumab & pertuzumab & paclitaxel | Nothing  v taxanes | Trastuzumab & pertuzumab | Data extracted but not included in published analyses |
| NCT01428414 | [73] | 1. Trastuzumab & paclitaxel & carboplatin  2. Trastuzumab & paclitaxel & epirubicin | Taxane & carboplatin  v  Taxane & epirubicin |  | Data extracted but not included in published analyses |
| JO17360 | [74] | 1. Docetaxel & trastuzumab  2. Trastuzumab (then docetaxel & trastuzumab – AEs reported separately) | Taxanes  v  Nothing |  | Data extracted but not included in published analyses |
| Robert 2006 | [75] | 1. Paclitaxel & trastuzumab  2. Paclitaxel & carboplatin & trastuzumab | Taxanes  v taxanes & carboplatin |  | Data extracted but not included in published analyses |
| HERNATA  NCT00430001 | [76] | 1. Docetaxel & trastuzumab  2. Vinorelbine & trastuzumab | Taxanes  v vinorelbine |  | Data extracted but not included in published analyses |
| BCIRG-007  NCT00047255 | [77] | 1. Docetaxel & trastuzumab  2. Docetaxel & carboplatin & trastuzumab  Prior trastuzumab had to be more than 12 months before, prior chemotherapy more than 6 months before. | Taxanes  v  Taxanes & carboplatin |  | Data extracted but not included in published analyses |
| STM01-102  NCT00294996 | [78] | 1. NPLD (doxorubicin) & paclitaxel & trastuzumab  2. Paclitaxel & trastuzumab  A third had prior anthracycline treatment | Taxanes |  | Data extracted but not included in published analyses |
| TRAVIOTA | [79] | 1. Vinorelbine & trastuzumab  2. Paclitaxel & trastuzumab  3. Docetaxel & trastuzumab  Any prior trastuzumab had to be more than 12 months previously, no other use of study drugs | Vinorelbine  v  Taxanes |  | Data extracted but not included in published analyses |
| CHAT  MO16419  NCT02748213 | [80,81] | 1. Docetaxel & capecitabine & trastuzumab  2. Docetaxel & trastuzumab | Taxanes  v  Taxanes & capecitabine |  | Data extracted but not included in published analyses |
| TRYPHAENA | [82] | 1. FEC & trastuzumab & pertuzumab then docetaxel (neoadjuvant), trastuzumab (adjuvant)  2. FEC then docetaxel & trastuzumab & pertuzumab (neoadjuvant), trastuzumab (adjuvant)  3. Docetaxel & carboplatin & trastuzumab (neoadjuvant), trastuzumab (adjuvant)  Patients received other therapies per physician choice | FEC & taxanes  v  taxanes & carboplatin | Trastuzumab  v trastuzumab & pertuzumab | Data extracted but not included in published analyses  Took the neoadjuvant adverse events only. |
| NCT00201760 | [83] | 1. Gemcitabine, cisplatin, trastuzumab 2. Gemcitabine, trastuzumab | Gemcitabine & cisplatin  v  Gemcitabine |  | Not included in published analyses |
| NCT00976989 | [84] | 1. Neoadjuvant 5-Fluorouracil, epirubicin with cyclophosphamide (FEC), trastuzumab and pertuzumab followed by docetaxel, trastuzumab and pertuzumab, then trastuzumab adjuvant 2. Neoadjuvant 5-Fluorouracil, epirubicin with cyclophosphamide (FEC), followed by docetaxel, trastuzumab and pertuzumab, then trastuzumab adjuvant 3. Neoadjuvant trastuzumab, carboplatin, docetaxel (TCH) and pertuzumab then trastuzumab adjuvant | T+P Concomitant Anthracycline-based Chemotherapy  v  T+P Sequential Anthracycline-based Chemotherapy  v  T+P Concomitant Non-Anthracycline Chemotherapy |  | Not included in published analysis – adverse events not reported separately for neoadjuvant period |

RCTs comparing different doses/lengths of trastuzumab

| **Trial name(s)** | **Key reference** | **Drug regimes in each arm** | **Chemo regimes** | **Other HER2 drugs** | **Included/excluded?** |
| --- | --- | --- | --- | --- | --- |
| PHARE  NCT00381901 | [86,87] | 1. 12 months trastuzumab  2. 6 months trastuzumab  Mixed chemotherapies  (radiotherapy & hormone therapy allowed) | Mixed |  | Data extracted but not included in published analyses |
| E-2198  NCT00003992 | [88] | 1. paclitaxel & trastuzumab (TH) then doxorubicin & cyclophosphamide (AC)  2. paclitaxel & trastuzumab (TH) then doxorubicin & cyclophosphamide (AC) then 12 months trastuzumab  (radiotherapy & hormone therapy allowed) | Taxanes & AC |  | Data extracted but not included in published analyses |
| PERSEPHONE  NCT00712140 | [89,90] | 1. 12 months trastuzumab  2. 6 months trastuzumab  Mixed chemotherapies  (radiotherapy & hormone therapy allowed) | Mixed |  | Data extracted but not included in published analyses |
| HORG  NCT00615602 | [91] | 1. FEC then 12 months docetaxel & trastuzumab  2. FEC then 6 months docetaxel & trastuzumab | FEC & taxanes |  | Data extracted but not included in published analyses |
| HELEX  NCT00999804 | [92] | 1. Lapatinib & trastuzumab (24 weeks)  2. Lapatinib & trastuzumab (12 weeks)  (hormone therapy allowed) | None |  | Data extracted but not included in published analyses |
| H0650g | [93] | 1. Trastuzumab (4mg/kg then 2mg/kg)  2. Trastuzumab (8mg/kg then 4mg/kg)  No previous chemotherapy, no concurrent radiotherapy |  |  | Included in paper, not in meta-analysis |

RCTs with trastuzumab in both arms but other modifications (eg. timing, addition of another kind of drug)

| **Trial name(s)** | **Key reference** | **Drug regimes in each arm** | **Chemo regimes** | **Other HER2 drugs** | **Included/excluded?** |
| --- | --- | --- | --- | --- | --- |
| ACOSOG Z1041  NCT00513292 | [94,95] | 1: FEC then trastuzumab & paclitaxel  2: paclitaxel & trastuzumab then FEC & trastuzumab | FEC & taxanes |  | Data extracted but not included in published analyses |
| APHINITY  BIG 4−11  NCT01358877 | [96,97] | 1. chemotherapy & trastuzumab & pertuzumab  2. chemotherapy & trastuzumab & placebo | mixed | Trastuzumab  v trastuzumab & pertuzumab | Data extracted but not included in published analyses |
| CLEOPATRA  NCT00567190 | [98,99] | 1. Docetaxel & trastuzumab & pertuzumab  2. Docetaxel & trastuzumab & placebo  No hormone therapy allowed | Taxanes | Trastuzumab  v trastuzumab & pertuzumab | Data extracted but not included in published analyses |
| BOLERO-1  NCT00876395 | [100,101] | 1. Paclitaxel & trastuzumab & everolimus  2. Paclitaxel & trastuzumab & placebo  No hormone therapy allowed | Taxanes  v  Taxanes & mTor inhibitor |  | Data extracted but not included in published analyses |
| ECOG E1105  NCT00520975 | [102] | 1. Paclitaxel & carboplatin & trastuzumab & bevacizumab  2. Paclitaxel & carboplatin & trastuzumab & placebo | Taxanes & carboplatin | Trastuzumab  v trastuzumab & bevacizumab | Data extracted but not included in published analyses |
| AVEREL  NCT00391092 | [103,104] | 1. Docetaxel & trastuzumab  2. Docetaxel & trastuzumab & bevacizumab | Taxanes | Trastuzumab  v trastuzumab & bevacizumab | Data extracted but not included in published analyses |
| NSABP FB-7  NCT01008150 | [105,106] | 1. Paclitaxel & trastuzumab then doxorubicin & cyclophosphamide (neoadjuvant), trastuzumab (adjuvant)  2. Paclitaxel & neratinib then doxorubicin & cyclophosphamide (neoadjuvant), trastuzumab (adjuvant)  3. Paclitaxel & trastuzumab & neratinib then doxorubicin & cyclophosphamide (neoadjuvant), trastuzumab (adjuvant)  All arms had trastuzumab as adjuvant therapy | Taxanes & AC | Trastuzumab v  trastuzumab & neratinib | Data extracted but not included in published analyses |
| PHEREXA  NCT01026142 | [107] | 1. Capecitabine & trastuzumab  2. Capecitabine & trastuzumab & pertuzumab  No other concurrent treatments | Capecitabine | Trastuzumab  v trastuzumab & pertuzumab | Data extracted but not included in published analyses |
| PERTAIN  NCT01491737 | [108,109] | 1. Optional chemotherapy & trastuzumab  2. Optional chemotherapy & trastuzumab & pertuzumab  All had AI hormone therapy | Docetaxel/paclitaxel | Trastuzumab  v trastuzumab & pertuzumab | Data extracted but not included in published analyses |
| BOLERO-3  NCT01007942 | [101,110] | 1. Everolimus & vinorelbine & trastuzumab  2. Placebo & vinorelbine & trastuzumab  All patients had previously had taxane-based chemotherapy & trastuzumab | mTor inhibitor & vinorelbine  v  vinorelbine |  | Data extracted but not included in published analyses |
| HER2CLIMB  NCT02614794 | [111] | 1. Tucatinib, capecitabine, trastuzumab 2. Placebo, capecitabine, trastuzumab | Tucanib & capecitabine  v  placebo & capecitabine |  | Not included in published analyses |
| PEONY  NCT02586025 | [112] | 1. Pertuzumab, docetaxel & trastuzumab 2. Placebo, docetaxel & trastuzumab | Docetaxel & pertuzumab  v docetaxel & placebo |  | Not included in published analyses |
| NCT02586025 | [85] | 1. Trastuzumab, docetaxel & pertuzumab 2. Trastuzumab, docetaxel & placebo | Taxanes | Trastuzumab  v trastuzumab & pertuzumab | Not included in published analysis |

Others

| LUX-Breast1  NCT01125566 | [113,114] | 1. Afatinib & vinorelbine  2. Trastuzumab & vinorelbine  All patients previously treated with trastuzumab | Vinorelbine | Trastuzumab  v afatinib | Data extracted but not included in published analyses: comparison/combination with other experimental drug |
| --- | --- | --- | --- | --- | --- |
| SOPHIA  NCT02492711 | [115] | 1. Chemotherapy & margetuximab 2. Chemotherapy & trastuzumab   Chemotherapy was of physician’s choice |  | Trastuzumab  v margetuximab | Not included in published analysis: comparison with other experimental drug |
| NCT00545688 | [116] | 1. Neoadjuvant docetaxel & trastuzumab 2. Neoadjuvant docetaxel & pertuzumab & trastuzumab 3. Neoadjuvant pertuzumab & trastuzumab 4. Neoadjuvant docetaxel & pertuzumab   All patients received adjuvant trastuzumab |  |  | Not included in published analysis: adverse events not recorded for neoadjuvant period alone |
| **NEFERTT**  NCT00915018 | [117] | 1. Paclitaxel & neratinib 2. Paclitaxel & trastuzumab | Taxanes | Trastuzumab  v  neratinib | Not included in published analysis: comparison with other experimental drug |
| **TEAL** | [118] | 1. Ado-trastuzumab emtansine (T-DM1), lapatinib & nab-paclitaxel 2. trastuzumab, pertuzumab, and paclitaxel |  |  | Not included in published analysis: arms not comparable |

RCTs excluded during search

| **Trial name(s)** | **Drug regimes in each arm** | | **Chemo regimes** | **Other HER2 drugs** | | **Included/excluded?** |  |
| --- | --- | --- | --- | --- | --- | --- | --- |
| JBCRG-10  [119] | 1. FEC then docetaxel & trastuzumab (TCH)  2. Docetaxel & trastuzumab (TCH) then FEC  3. Docetaxel & trastuzumab (TCH) | | FEC & taxanes & carboplatin |  | | Adverse events not reported |  |
| N-SAS BC 07  RESPECT  NCT01104935  [120] | 1. 12 months trastuzumab  2. chemotherapy (any) & 12 months trastuzumab  Radiotherapy & hormone therapy allowed | | mixed |  | | Adverse events not reported |  |
| Short-HER  NCT00629278  [121] | 1. AC or EC then docetaxel & trastuzumab (14 weeks)  2. docetaxel & trastuzumab (3 weeks) then FEC  (hormone & radiotherapy allowed) | | AC or EC & taxanes  v  FEC & taxanes | 14 weeks v 3 weeks trastuzumab | | Adverse events not reported (apart from cardiac events) |  |
| SOLD  NCT00593697  [122] | 1. docetaxel plus trastuzumab (9 weeks) then FEC  2. docetaxel plus trastuzumab (9 weeks) then FEC and 12 months trastuzumab | | Taxanes & FEC | 9 week trastuzumab v 12 month trastuzumab | | Excluded – adverse events only reported during chemotherapy (when two groups identical), apart from cardiac events |  |
| HERCULES | 3 arms, but differing in HER2 status. Comparable arms not differing in trastuzumab regime | |  |  | | Not included because arms not randomised or properly comparable |  |
| TH3RESA  NCT01419197  [123,124] | 1. Trastuzumab emtansine (T-DM1) 2. Chemotherapy only (physician’s choice)   All patients had previously had taxane and at least two HER-2 directed therapies | | Mixed chemotherapy regimes | T-DM1  v chemotherapy only | | Not included because control group is physician’s choice. |  |
| HERTAX  [125] | 1. Docetaxel and trastuzumab  2. Trastuzumab (then docetaxel after disease progression)  Previous non-taxane chemotherapy allowed | | Taxanes |  | | Not included because adverse events summarise all, including after arm 2 moved to docetaxel. |  |
| Storniolo 2009 | Dose escalation study of lapatinib with arms with/without trastuzumab | |  |  | | Not included because of dose changes |  |
| Andre 2010  [110] | Dose escalation study of everolimus with paclitaxel & trastuzumab | |  |  | | Not included because of dose changes |  |
| NCT00674414 | 1. Neoadjuvant trastuzumab  2. Neoadjuvant trastuzumab & everolimus | | mTor inhibitor  v  none |  | | Terminated and no results published |  |
| NCT01304784 | Drug MM-111 & trastuzumab in combinations with various other regimes | |  |  | | Not included because of confounding factor of new drug MM-111 |  |
| LILAC  NCT01901146  [126] | Drug ABP 980 & trastuzumab in various combinations and doses | |  |  | | Not included because of confounding factor of new drug ABP 980 |  |
| LPT112515  NCT00968968  [127] | 1. Paclitaxel (80mg) & trastuzumab & lapatinib (1000mg)  2. Paclitaxel (70mg) & trastuzumab & lapatinib (1000mg)  3. Paclitaxel (80mg) & trastuzumab & lapatinib (750mg) | |  |  | | Not included because of dose changes in concomitant treatments in different arms |  |
| NCT01526369 | 1. Paclitaxel & trastuzumab  2. Paclitaxel & trastuzumab & lapatinib | | Taxanes | Trastuzumab v trastuzumab & lapatinib | | No data available as yet |  |
| NCT01471847 | 1. Drug BEZ235 & trastuzumab  2. Capecitabine & lapatinib | |  |  | | Not included because of confounding factor of new drug BEZ235 |  |
| NCT00963547  [128] | 1. Drug MK-2206 (45mg) & trastuzumab  2. Drug MK-2206 (60mg) & trastuzumab  3. Drug MK-2206 (135mg) & trastuzumab  Drug MK-2206 (200mg) & trastuzumab | |  |  | | Not included because of confounding factor of new drug MK-2206 |  |
| EGF104383  NCT00272987  [129] |  | |  |  | | Excluded: dose-escalation study |  |
| Chang 2010  [17] | 1. Neoadjuvant docetaxel & carboplatin 2. Neoadjuvant docetaxel, carboplatin & trastuzumab | | Docetaxel & carboplatin | Trastuzumab  v nothing | | Adverse effects only reported as ‘similar’ in two groups and percentages given for all patients combined. |  |
| DESTINY  NCT03529110  [130] | 1. Trastuzumab Deruxtecan (T-DXd) 2. Ado-trastuzumab Emtansine (T-DM1) | |  | T-DXd  v T-DM1 | | Excluded because of comparison with T-DXd |  |
| KATE2  NCT02924883  [131] | 1. Trastuzumab Emtansine (T-DM1) & Atezolizumub 2. Trastuzumab Emtansine (T-DM1) & placebo | |  | Atezolizumub and T-DM1  v T-DM1 | | Excluded because comparison is Atezolizumub v placebo |  |
| Holmes 2011  [132] | 1. Neoadjuvant trastuzumab 2. Neoadjuvant trastuzumab & lapatinib 3. Neoadjuvant lapatinib   All patients then got neoadjuvant FEC and paclitaxel | |  | Trastuzumab  v trastuzumab & lapatinib  v lapatinib | | No adverse events recorded |  |
| Zhou 2016  [133] | 1. Neoadjuvant docetaxel, carboplatin, trastuzumab (TCH) 2. Neoadjuvant docetaxel, epirubicin, cyclophosphamide (TAC) | Carboplatin  v  Epirubicin & cyclophosphamide | | Trastuzumab  v  Nothing | No adverse event reporting | | |
| NCT00444587 | 1. Continue on chemotherapy & trastuzumab 2. Continue on chemotherapy only   All patients were already taking chemotherapy & trastuzumab | Chemotherapy of physician’s choice | |  | Not included as patients were already on trastuzumab and chemotherapy was physicians’ choice | | |
| NCT01998906  [134] | 1. Neoadjuvant doxorubicin, paclitaxel; then cyclophosphamide, methotrexate, fluorouracil (CMF) all with trastuzumab and adjuvant trastuzumab & tamoxifen for HER2+ patients 2. Neoadjuvant doxorubicin, paclitaxel; then cyclophosphamide, methotrexate, fluorouracil (CMF) for HER2+ patients 3. Neoadjuvant doxorubicin, paclitaxel; then cyclophosphamide, methotrexate, fluorouracil (CMF) for HER2- patients | Taxanes & CMF | | Trastuzumab  v  No trastuzumab | Not included as trastuzumab patients also given tamoxifen after surgery and adverse events not recorded for (comparable) neoadjuvant period separately. | | |
| GeparQuattro  GBG 40  NCT00288002  [135] | 1. Docetaxel 2. Docetaxel & capecitabine together 3. Docetaxel then capecitabine   Trastuzumab given to HER2+ patients within each arm |  | |  | Not included: adverse events not reported separately for the different chemotherapy arms, only as ‘chemotherapy’ and ‘chemotherapy plus trastuzumab’ grouping | | |
| Precious  JBCRC M05  [136] | 1. Chemotherapy of physician’s choice, pertuzumab & trastuzumab 2. Chemotherapy of physician’s choice & trastuzumab |  | |  | Not included – chemotherapy was ‘physicians’ choice’ | | |

## References

1. Azambuja E De *et al.* 2014 Trastuzumab-Associated Cardiac Events at 8 Years of Median Follow-Up in the Herceptin Adjuvant Trial ( BIG. *J. Clin. Oncol.* **32**, 2159–2165. (doi:10.1200/JCO.2013.53.9288)

2. Gianni L *et al.* 2011 Treatment with trastuzumab for 1 year after adjuvant chemotherapy in patients with HER2-positive early breast cancer : a 4-year follow-up of a randomised controlled trial. *Lancet Oncol.* **12**, 236–244. (doi:10.1016/S1470-2045(11)70033-X)

3. Cameron D *et al.* 2017 11 years’ follow-up of trastuzumab after adjuvant chemotherapy in HER2-positive early breast cancer: final analysis of the HERceptin Adjuvant ( HERA ) trial. *Lancet* **389**, 1195–1205. (doi:10.1016/S0140-6736(16)32616-2)

4. Hoffmann-La Roche. 2017 NCT00045032 Clinical registry results: Herceptin (Trastuzumab) in Treating Women With Human Epidermal Growth Factor Receptor (HER) 2-Positive Primary Breast Cancer (HERA). *ClinicalTrials.gov*. See https://clinicaltrials.gov/ct2/show/results/NCT00045032.

5. Perez EA *et al.* 2011 Four-Year Follow-Up of Trastuzumab Plus Adjuvant Chemotherapy for Operable Human Epidermal Growth Factor Receptor 2 – Positive Breast Cancer : Joint Analysis of Data From NCCTG N9831 and NSABP B-31. *J. Clin. Oncol.* **29**, 3366–3373. (doi:10.1200/JCO.2011.35.0868)

6. Romond EH *et al.* 2005 Trastuzumab plus Adjuvant Chemotherapy for Operable HER2-Positive Breast Cancer. *N. Engl. J. Med.* **353**, 1673–1684.

7. Perez EA *et al.* 2011 Sequential Versus Concurrent Trastuzumab in Adjuvant Chemotherapy for Breast Cancer. *J. Clin. Oncol.* **29**, 4491–4497. (doi:10.1200/JCO.2011.36.7045)

8. Advani PP, Ballman K V, Dockter TJ, Colon-otero G, Perez EA, Advani PP, Colon-otero G. 2016 Long-Term Cardiac Safety Analysis of NCCTG N9831 ( Alliance ) Adjuvant Trastuzumab Trial. *J. Clin. Oncol.* **34**, 581–587. (doi:10.1200/JCO.2015.61.8413)

9. Slamon D *et al.* 2011 Adjuvant Trastuzumab in HER2-Positive Breast Cancer. *N. Engl. J. Med.* **365**, 1273–1283. (doi:10.1056/NEJMoa0910383)

10. Sanofi. 2016 NCT00021255 clinical trials registry results: Combination Chemotherapy With or Without Trastuzumab in Treating Women With Breast Cancer. *ClinicalTrials.gov*. See https://clinicaltrials.gov/ct2/show/results/NCT00021255.

11. Spielmann M *et al.* 2009 Trastuzumab for Patients With Axillary-Node – Positive Breast Cancer : Results of the FNCLCC-PACS 04 Trial. *J. Clin. Oncol.* **27**, 6129–6134. (doi:10.1200/JCO.2009.23.0946)

12. Gianni L *et al.* In press. Neoadjuvant chemotherapy with trastuzumab followed by adjuvant trastuzumab versus neoadjuvant chemotherapy alone , in patients with HER2-positive locally advanced breast cancer ( the NOAH trial ): a randomised controlled superiority trial with a parallel . *Lancet* **375**, 377–384. (doi:10.1016/S0140-6736(09)61964-4)

13. Huober J *et al.* 2012 Higher efficacy of letrozole in combination with trastuzumab compared to letrozole monotherapy as first-line treatment in patients with HER2-positive, hormone-receptor-positive metastatic breast cancer - Results of the eLEcTRA trial. *Breast* **21**, 27–33. (doi:10.1016/j.breast.2011.07.006)

14. Gasparini G *et al.* 2007 Randomized Phase II Trial of weekly paclitaxel alone versus trastuzumab plus weekly paclitaxel as first-line therapy of patients with Her-2 positive advanced breast cancer. *Breast Cancer Res. Treat.* **101**, 355–365. (doi:10.1007/s10549-006-9306-9)

15. Slamon DJ *et al.* 2001 Use of chemotherapy plus a monoclonal antibody against HER2 for metastatic breast cancer that overexpresses HER2. *N. Engl. J. Med.* **344**, 783–92. (doi:10.1056/NEJM200103153441101)

16. Buzdar AU *et al.* 2005 Significantly higher pathologic complete remission rate after neoadjuvant therapy with trastuzumab, paclitaxel, and epirubicin chemotherapy: Results of a randomized trial in human epidermal growth factor receptor 2-positive operable breast cancer. *J. Clin. Oncol.* **23**, 3676–3685. (doi:10.1200/JCO.2005.07.032)

17. Chang HR, Glaspy J, Allison MA, Kass FC, Elashoff R, Chung DU, Gornbein J. 2010 Differential response of triple-negative breast cancer to a docetaxel and carboplatin-based neoadjuvant treatment. *Cancer* **116**, 4227–4237. (doi:10.1002/cncr.25309)

18. Jonsson Comprehensive Cancer Center. 2017 NCT00068341 clinical trials registry results: Docetaxel and Carboplatin With or Without Trastuzumab Before Surgery in Treating Women With Locally Advanced Breast Cancer. *ClinicalTrials.gov*. See https://clinicaltrials.gov/ct2/show/results/NCT00068341.

19. Seidman AD *et al.* 2008 Randomized phase III trial of weekly compared with every-3-weeks paclitaxel for metastatic breast cancer, with trastuzumab for all HER-2 overexpressors and random assignment to trastuzumab or not in HER-2 nonoverexpressors: Final results of cancer and leu. *J. Clin. Oncol.* **26**, 1642–1649. (doi:10.1200/JCO.2007.11.6699)

20. Steger GG *et al.* 2014 Epirubicin and docetaxel with or without capecitabine as neoadjuvant treatment for early breast cancer: Final results of a randomized phase III study (ABCSG-24). *Ann. Oncol.* **25**, 366–371. (doi:10.1093/annonc/mdt508)

21. Pierga J-Y *et al.* 2010 A multicenter randomized phase II study of sequential epirubicin / cyclophosphamide followed by docetaxel with or without celecoxib or trastuzumab according to HER2 status, as primary chemotherapy for localized invasive breast cancer patients. *Breast Cancer Res Treat* **122**, 429–437. (doi:10.1007/s10549-010-0939-3)

22. Kaufman B *et al.* 2009 Trastuzumab plus anastrozole versus anastrozole alone for the treatment of postmenopausal women with human epidermal growth factor receptor 2-positive, hormone receptor-positive metastatic breast cancer: Results from the randomized phase III TAnDEM study. *J. Clin. Oncol.* **27**, 5529–5537. (doi:10.1200/JCO.2008.20.6847)

23. Hoffmann-La Roche. 2013 NCT00022672 clinical trial registry results: A Study to Evaluate the Efficacy and Safety of Herceptin® (Trastuzumab) in Combination With Arimidex® (Anastrozole) an Aromatase Inhibitor Compared to Arimidex® Alone in Patients With Metastatic Breast Cancer. *ClinicalTrials.gov*.

24. Marty M *et al.* 2005 Randomized phase II trial of the efficacy and safety of trastuzumab combined with docetaxel in patients with human epidermal growth factor receptor 2-positive metastatic breast cancer administered as first-line treatment: The M77001 study group. *J. Clin. Oncol.* **23**, 4265–4274. (doi:10.1200/JCO.2005.04.173)

25. Untch M, Loibl S, Bischoff J, Eidtmann H, Kaufmann M, Blohmer J, Hilfrich J, Strumberg D. 2012 Lapatinib versus trastuzumab in combination with neoadjuvant anthracycline-taxane-based chemotherapy (GeparQuinto, GBG 44): a randomised phase 3 trial. *Lancet* **13**, 135–144. (doi:10.1016/S1470-2045(11)70397-7)

26. Kokko R, Hemminki A, Tarkkanen M, Jääskeläinen A, Pajunen M, Rauhala M. 2006 Adjuvant Docetaxel or Vinorelbine with or without Trastuzumab for Breast Cancer. *N. Engl. J. Med.* **354**, 809–820. (doi:10.1056/NEJMoa053028)

27. Von Minckwitz G *et al.* 2011 Trastuzumab beyond progression: Overall survival analysis of the GBG 26/BIG 3-05 phase III study in HER2-positive breast cancer. *Eur. J. Cancer* **47**, 2273–2281. (doi:10.1016/j.ejca.2011.06.021)

28. Von Minckwitz G *et al.* 2009 Trastuzumab beyond progression in human epidermal growth factor receptor 2-positive advanced breast cancer: A German Breast Group 26/Breast International Group 03-05 study. *J. Clin. Oncol.* **27**, 1999–2006. (doi:10.1200/JCO.2008.19.6618)

29. Minckwitz G Von *et al.* 2008 Capecitabine vs. capecitabine + trastuzumab in patients with HER2-positive metastatic breast cancer progressing during trastuzumab treatment: The TBP phase III study (GBG 26/BIG 3–05). *J. Clin. Oncol.* **26**, 1025–1025. (doi:10.1200/jco.2008.26.15_suppl.1025)

30. National Cancer Institute (NCI). 2020 NCT01275677 clinical trial registry results: Chemotherapy With or Without Trastuzumab After Surgery in Treating Women With Invasive Breast Cancer. *ClinicalTrials.gov*. See https://clinicaltrials.gov/ct2/show/results/NCT01275677.

31. Fehrenbacher L *et al.* 2020 NSABP B-47/NRG oncology phase III randomized trial comparing adjuvant chemotherapy with or without trastuzumab in high-risk invasive breast cancer negative for HER2 by FISH and with IHC 1+ or 2+. *J. Clin. Oncol.* **38**, 444–453. (doi:10.1200/JCO.19.01455)

32. National Cancer Institute (NCI). 2022 NCT00769379 clinical trial registry results: Radiation Therapy With or Without Trastuzumab in Treating Women With Ductal Carcinoma In Situ Who Have Undergone Lumpectomy. *ClinicalTrials.gov*. See https://clinicaltrials.gov/ct2/show/NCT00769379.

33. Siziopikou KP *et al.* 2013 Preliminary results of centralized HER2 testing in ductal carcinoma in situ (DCIS): NSABP B-43. *Breast Cancer Res. Treat.* **142**, 415–421. (doi:10.1007/s10549-013-2755-z)

34. Sidney Kimmel Comprehensive Cancer Center at Johns Hopkins. 2019 NCT00971737 clinical trial registry results: Cyclophosphamide and Vaccine Therapy With or Without Trastuzumab in Treating Patients With Metastatic Breast Cancer. *ClinicalTrials.gov*. See https://clinicaltrials.gov/ct2/show/results/NCT00971737.

35. Hurvitz S *et al.* 2013 Final analysis of a phase II 3-arm randomized trial of neoadjuvant trastuzumab or lapatinib or the combination of trastuzumab and lapatinib, followed by six cycles of docetaxel and carboplatin with trastuzumab and/or lapatinib in patients with HER2+ breas. *Cancer Res.* **73**, Abstract S1-02. (doi:10.1158/0008-5472.SABCS13-S1-02)

36. Novartis Pharmaceuticals. 2014 NCT00490139 clinical trials registry results: ALTTO (Adjuvant Lapatinib And/Or Trastuzumab Treatment Optimisation) Study; BIG 2-06/N063D (ALTTO). *ClinicalTrials.gov*. See https://clinicaltrials.gov/ct2/show/results/NCT00490139.

37. Spanish Breast Cancer Research Group. 2018 NCT00841828 clinical trial registry results: Trastuzumab Versus Lapatinib as Neoadjuvant Treatment for Her2+ Patients. *ClinicalTrials.gov*. See https://clinicaltrials.gov/ct2/show/results/NCT00841828.

38. Bonnefoi H *et al.* 2015 Neoadjuvant treatment with docetaxel plus lapatinib, trastuzumab, or both followed by an anthracycline-based chemotherapy in HER2-positive breast cancer: Results of the randomised phase II EORTC 10054 study. *Ann. Oncol.* **26**, 325–332. (doi:10.1093/annonc/mdu551)

39. Carey LA *et al.* 2016 Molecular heterogeneity and response to neoadjuvant human epidermal growth factor receptor 2 targeting in CALGB 40601, a randomized phase III trial of paclitaxel plus trastuzumab with or without lapatinib. *J. Clin. Oncol.* **34**, 542–549. (doi:10.1200/JCO.2015.62.1268)

40. National Cancer Institute (NCI). 2015 NCT00770809 clinical trial registry results: Paclitaxel and Trastuzumab With or Without Lapatinib in Treating Patients With Stage II or Stage III Breast Cancer That Can Be Removed by Surgery. *ClinicalTrials.gov*. See https://clinicaltrials.gov/ct2/show/results/NCT00770809.

41. Gelmon KA *et al.* 2015 Lapatinib or trastuzumab plus taxane therapy for human epidermal growth factor receptor 2-positive advanced breast cancer: Final results of NCIC CTG MA.31. *J. Clin. Oncol.* **33**, 1574–1583. (doi:10.1200/JCO.2014.56.9590)

42. Novartis Pharmaceuticals. 2014 NCT00667251 clinical trials registry results: Chemotherapy and Lapatinib or Trastuzumab in Treating Women With HER2/Neu-Positive Metastatic Breast Cancer. *ClinicalTrials.gov*. See https://clinicaltrials.gov/ct2/show/results/NCT00667251.

43. Pivot X *et al.* 2015 CEREBEL (EGF111438): A phase III, randomized, open-label study of lapatinib plus capecitabine versus trastuzumab plus capecitabine in patients with human epidermal growth factor receptor 2-positive metastatic breast cancer. *J. Clin. Oncol.* **33**, 1564–1573. (doi:10.1200/JCO.2014.57.1794)

44. Novartis Pharmaceuticals. 2013 NCT00820222 clinical trial registry results: Lapatinib Plus Capecitabine Versus Trastuzumab Plus Capecitabine in ErbB2 (HER2) Positive Metastatic Breast Cancer. *ClinicalTrials.gov*. See https://clinicaltrials.gov/ct2/show/results/NCT00820222.

45. Guarneri V *et al.* 2012 Preoperative chemotherapy plus trastuzumab, lapatinib, or both in human epidermal growth factor receptor 2-positive operable breast cancer: Results of the randomized phase II CHER-LOB study. *J. Clin. Oncol.* **30**, 1989–1995. (doi:10.1200/JCO.2011.39.0823)

46. GlaxoSmithKline. 2016 NCT00429299 clinical trial registry results: Neoadjuvant Study With Chemotherapy, Lapatinib And Trastuzumab In Breast Cancer (CHERLOB). *ClinicalTrials.gov*.

47. Robidoux A *et al.* 2013 Lapatinib as a component of neoadjuvant therapy for HER2-positive operable breast cancer ( NSABP protocol B-41 ): an open-label , randomised phase 3 trial. *Lancet Oncol.* **14**, 1183–1192. (doi:10.1016/S1470-2045(13)70411-X)

48. Baselga J, Bradbury I, Eidtmann H, Cosimo S Di, Azambuja E De, Aura C, Gómez H, Dinh P, Fauria K. 2012 Lapatinib with trastuzumab for HER2-positive early breast cancer ( NeoALTTO): a randomised, open-label, multicentre, phase 3 trial. *Lancet* **379**, 633–640. (doi:10.1016/S0140-6736(11)61847-3)

49. Salgado R *et al.* 2015 Tumor-Infiltrating Lymphocytes and Associations With Pathological Complete Response and Event-Free Survival in HER2-Positive Early-Stage Breast Cancer Treated With Lapatinib and Trastuzumab: A Secondary Analysis of the NeoALTTO Trial. *JAMA Oncol.* **1**, 448–455. (doi:10.1001/jamaoncol.2015.0830)

50. Azim Jr HA *et al.* 2013 Pattern of Rash, Diarrhea, and Hepatic Toxicities Secondary to Lapatinib and Their Association With Age and Response to Neoadjuvant Therapy: Analysis From the NeoALTTO Trial. *J. Clin. Oncol.* **31**. (doi:10.1200/JCO.2013.50.9448)

51. Johnston SRD *et al.* 2018 Phase III, Randomized study of dual human epidermal growth factor receptor 2 (HER2) blockade with lapatinib plus trastuzumab in combination with an aromatase inhibitor in postmenopausal women with HER2-positive, hormone receptor-positive metastatic breast. *J. Clin. Oncol.* **36**, 741–748. (doi:10.1200/JCO.2017.74.7824)

52. Novartis Pharmaceuticals. 2019 NCT01160211 clinical trial registry results: A Study to Compare the Safety and Efficacy of an Aromatase Inhibitor in Combination With Lapatinib, Trastuzumab or Both for the Treatment of Hormone Receptor Positive, HER2+ Metastatic Breast Cancer. *ClinicalTrials.gov*. See https://clinicaltrials.gov/ct2/show/results/NCT01160211.

53. GlaxoSmithKline. 2011 NCT00524303 clinical trial registry results: Lapatinib +/- Trastuzumab In Addition To Standard Neoadjuvant Breast Cancer Therapy. *ClinicalTrials.gov*. See https://clinicaltrials.gov/ct2/show/results/NCT00524303.

54. GlaxoSmithKline. 2013 LPT109096 Clinical Study Report.

55. Blackwell KL *et al.* 2012 Overall survival benefit with lapatinib in combination with trastuzumab for patients with human epidermal growth factor receptor 2-positive metastatic breast cancer: Final results from the EGF104900 study. *J. Clin. Oncol.* **30**, 2585–2592. (doi:10.1200/JCO.2011.35.6725)

56. GlaxoSmithKline. 2011 NCT00320385 clinical trial registry results: Lapatinib In Combination With Trastuzumab Versus Lapatinib Monotherapy In Subjects With HER2-positive Metastatic Breast Cancer. *ClinicalTrials.gov*. See https://clinicaltrials.gov/ct2/show/results/NCT00320385.

57. Takano T *et al.* 2018 A randomized phase II trial of trastuzumab plus capecitabine versus lapatinib plus capecitabine in patients with HER2-positive metastatic breast cancer previously treated with trastuzumab and taxanes: WJOG6110B/ELTOP. *Breast* **40**, 67–75. (doi:10.1016/j.breast.2018.04.010)

58. Nitz UA *et al.* 2017 De-escalation strategies in HER2-positive early breast cancer (EBC): Final analysis of the WSG-ADAPT HER2+/HR- phase II trial: Efficacy, safety, and predictive markers for 12 weeks of neoadjuvant dual blockade with trastuzumab and pertuzumab ± weekly pacl. *Ann. Oncol.* **28**, 2768–2772. (doi:10.1093/annonc/mdx494)

59. Harbeck N *et al.* 2017 De-escalation strategies in human epidermal growth factor receptor 2 (HER2)-positive early breast cancer (BC): Final analysis of the West German study group Adjuvant Dynamic Marker-Adjusted Personalized Therapy Trial Optimizing Risk Assessment and Therapy. *J. Clin. Oncol.* **35**, 3046–3054. (doi:10.1200/JCO.2016.71.9815)

60. Hoffmann-La Roche. 2017 NCT02131064 clinical trial registry results: A Study Evaluating Trastuzumab Emtansine Plus Pertuzumab Compared With Chemotherapy Plus Trastuzumab and Pertuzumab for Participants With Human Epidermal Growth Factor Receptor 2 (HER2)-Positive Breast Cancer. *ClinicalTrials.gov*. See https://clinicaltrials.gov/ct2/show/results/NCT02131064.

61. Hurvitz SA *et al.* 2013 Phase II Randomized Study of Trastuzumab Emtansine Versus Trastuzumab Plus Docetaxel in Patients With Human Epidermal Growth Factor Receptor 2 – Positive Metastatic Breast Cancer. *J. Clin. Oncol.* **31**, 1157–1164. (doi:10.1200/JCO.2012.44.9694)

62. Hoffmann-La Roche. 2013 NCT00679341 clinical trials registry entry: *ClinicalTrials.gov*. See https://clinicaltrials.gov/ct2/show/results/NCT00679341.

63. Hoffmann-La Roche. 2017 NCT01120184 clinical trial registry results: A Study of Trastuzumab Emtansine (T-DM1) Plus Pertuzumab/Pertuzumab Placebo Versus Trastuzumab [Herceptin] Plus a Taxane in Participants With Metastatic Breast Cancer (MARIANNE). *ClinicalTrials.gov*. See https://clinicaltrials.gov/ct2/show/results/NCT01120184.

64. Hoffmann-La Roche. 2019 NCT01772472 clinical trial registry results: A Study of Trastuzumab Emtansine Versus Trastuzumab as Adjuvant Therapy in Patients With HER2-Positive Breast Cancer Who Have Residual Tumor in the Breast or Axillary Lymph Nodes Following Preoperative Therapy . *ClinicalTrials.gov*.

65. Hoffmann-La Roche. 2013 NCT00829166 clinical trial registry results: A Study of Trastuzumab Emtansine Versus Capecitabine + Lapatinib in Participants With HER2-positive Locally Advanced or Metastatic Breast Cancer (EMILIA). *ClinicalTrials.gov*. See https://clinicaltrials.gov/ct2/show/results/NCT00829166.

66. Genentech Inc. 2016 NCT00951665 clinical trials registry entry: A Study of Trastuzumab Emtansine, Paclitaxel, and Pertuzumab in Patients With HER2-Positive, Locally Advanced or Metastatic Breast Cancer. *ClinicalTrials.gov*. See https://clinicaltrials.gov/ct2/show/NCT00951665.

67. Krop IE, Modi S, Lorusso PM, Pegram M, Guardino E, Althaus B, Lu D, Strasak A, Elias A. 2016 Phase 1b / 2a study of trastuzumab emtansine ( T-DM1 ), paclitaxel , and pertuzumab in HER2-positive metastatic breast cancer. *Breast Cancer Res.* , 1–10. (doi:10.1186/s13058-016-0691-7)

68. Dana-Farber Cancer Institute. 2022 NCT01853748 clinical trials registry results: T-DM1 vs Paclitaxel/Trastuzumab for Breast (ATEMPT Trial). *ClinicalTrials.gov*. See https://clinicaltrials.gov/ct2/show/NCT01853748.

69. Hatschek T *et al.* 2021 Neoadjuvant Trastuzumab, Pertuzumab, and Docetaxel vs Trastuzumab Emtansine in Patients with ERBB2-Positive Breast Cancer: A Phase 2 Randomized Clinical Trial. *JAMA Oncol.* **7**, 1360–1367. (doi:10.1001/jamaoncol.2021.1932)

70. Masuda N *et al.* 2020 A randomized, 3-arm, neoadjuvant, phase 2 study comparing docetaxel + carboplatin + trastuzumab + pertuzumab (TCbHP), TCbHP followed by trastuzumab emtansine and pertuzumab (T-DM1+P), and T-DM1+P in HER2-positive primary breast cancer. *Breast Cancer Res. Treat.* **180**, 135–146. (doi:10.1007/s10549-020-05524-6)

71. Hoffmann-La Roche. 2021 NCT01966471 clinical trial registry results: A Study of Trastuzumab Emtansine (Kadcyla) Plus Pertuzumab (Perjeta) Following Anthracyclines in Comparison With Trastuzumab (Herceptin) Plus Pertuzumab and a Taxane Following Anthracyclines as Adjuvant Therapy. See https://clinicaltrials.gov/ct2/show/results/NCT01966471.

72. Hoffmann-La Roche. 2017 NCT02144012 clinical trial registry results: A Study to Evaluate the Efficacy and Safety of Trastuzumab Emtansine Versus the Combination of Trastuzumab Plus Docetaxel in Patients With HER2-positive Breast Cancer. *ClinicalTrials.gov*. See https://clinicaltrials.gov/ct2/show/results/NCT02144012.

73. Huang L *et al.* 2015 Efficacy and safety analysis of trastuzumab and paclitaxel based regimen plus carboplatin or epirubicin as neoadjuvant therapy for clinical stage II-III, HER2-positive breast cancer patients: A phase 2, open-label, multicenter, randomized trial. *Oncotarget* **6**, 18683–18692. (doi:10.18632/oncotarget.4337)

74. Inoue K *et al.* 2010 Randomized phase III trial of trastuzumab monotherapy followed by trastuzumab plus docetaxel versus trastuzumab plus docetaxel as first-line therapy in patients with HER2-positive metastatic breast cancer: The JO17360 Trial Group. *Breast Cancer Res. Treat.* **119**, 127–136. (doi:10.1007/s10549-009-0498-7)

75. Robert N *et al.* 2006 Randomized Phase III Study of Trastuzumab , Paclitaxel , and Carboplatin Compared With Trastuzumab and Paclitaxel in Women With HER-2 – Overexpressing Metastatic Breast Cancer. *J. Clin. Oncol.* **24**, 2786–2792. (doi:10.1200/JCO.2005.04.1764)

76. Andersson M *et al.* 2011 Phase III randomized study comparing docetaxel plus trastuzumab with vinorelbine plus trastuzumab as first-line therapy of metastatic or locally advanced human epidermal growth factor receptor 2-positive breast cancer: The HERNATA study. *J. Clin. Oncol.* **29**, 264–271. (doi:10.1200/JCO.2010.30.8213)

77. Valero V *et al.* 2011 Multicenter phase III randomized trial comparing docetaxel and trastuzumab with docetaxel, carboplatin, and trastuzumab as first-line chemotherapy for patients with HER2-gene-amplified metastatic breast cancer (BCIRG 007 Study): Two highly active therapeu. *J. Clin. Oncol.* **29**, 149–156. (doi:10.1200/JCO.2010.28.6450)

78. Baselga J *et al.* 2014 Phase III trial of nonpegylated liposomal doxorubicin in combination with trastuzumab and paclitaxel in HER2-positive metastatic breast cancer. *Ann. Oncol.* **25**, 592–598. (doi:10.1093/annonc/mdt543)

79. Burstein HJ, Keshaviah A, Baron AD, Hart RD, Lambert-falls R, Marcom PK, Gelman R, Winer EP. 2007 Trastuzumab Plus Vinorelbine or Taxane Chemotherapy for HER2-overexpressing Metastatic Breast Cancer : The Trastuzumab and Vinorelbine or Taxane Study. *Cancer* **110**, 965–972. (doi:10.1002/cncr.22885)

80. Wardley AM *et al.* 2010 Randomized phase II trial of first-line trastuzumab plus docetaxel and capecitabine compared with trastuzumab plus docetaxel in HER2-positive metastatic breast cancer. *J. Clin. Oncol.* **28**, 976–983. (doi:10.1200/JCO.2008.21.6531)

81. Hoffmann-La Roche. 2016 NCT02748213 clinical trials registry results: A Study of Herceptin (Trastuzumab) in Women With Human Epidermal Growth Factor Receptor (HER) 2-Positive Advanced and/or Metastatic Breast Cancer. *ClinicalTrials.gov*. See https://clinicaltrials.gov/ct2/show/results/NCT02748213.

82. Schneeweiss A *et al.* 2013 Pertuzumab plus trastuzumab in combination with standard neoadjuvant anthracycline-containing and anthracycline-free chemotherapy regimens in patients with HER2-positive early breast cancer : a randomized phase II cardiac safety study ( TRYPHAENA ). *Ann. Oncol.* **24**, 2278–2284. (doi:10.1093/annonc/mdt182)

83. Kari Kendra Ohio State University Comprehensive Cancer Center. 2017 NCT00201760 clinical trial registry: Gemcitabine/ Trastuzumab and Gemcitabine/ Cisplatin/ Trastuzumab in Patients With Metastatic Breast Cancer. *ClinicalTrials.gov*. See https://clinicaltrials.gov/ct2/show/NCT00201760.

84. Hoffman-La Roche. 2016 NCT00976989 clinical trials registry results: A Study of Pertuzumab in Combination With Herceptin and Chemotherapy in Participants With HER2-Positive Breast Cancer. *ClinicalTrials.gov*. See https://clinicaltrials.gov/ct2/show/results/NCT00976989.

85. Hoffmann-La Roche. 2019 NCT02586025 clinical trial registry results: Study in Participants With Early-Stage or Locally Advanced Human Epidermal Growth Factor Receptor (HER) 2-Positive Breast Cancer to Evaluate Treatment With Trastuzumab Plus (+) Pertuzumab + Docetaxel Compared W. *ClinicalTrials.gov*. See https://clinicaltrials.gov/ct2/show/NCT02586025.

86. Pivot X *et al.* 2015 Cardiac toxicity events in the PHARE trial , an adjuvant trastuzumab randomised phase III study. *Eur. J. Cancer* **51**, 1660–1666. (doi:10.1016/j.ejca.2015.05.028)

87. Pivot X *et al.* 2013 6 months versus 12 months of adjuvant trastuzumab for patients with HER2-positive early breast cancer ( PHARE ): a randomised phase 3 trial. *Lancet Oncol.* **14**, 741–748. (doi:10.1016/S1470-2045(13)70225-0)

88. Schneider BP, O’Neill A, Shen F, Sledge GW, Thor AD, Kahanic SP, Zander PJ, Davidson NE. 2015 Pilot trial of paclitaxel-trastuzumab adjuvant therapy for early stage breast cancer: A trial of the ECOG-ACRIN cancer research group (E2198). *Br. J. Cancer* **113**, 1651–1657. (doi:10.1038/bjc.2015.405)

89. Earl HM *et al.* 2016 Trastuzumab-associated cardiac events in the Persephone trial. *Br. J. Cancer* **115**, 1462–1470. (doi:10.1038/bjc.2016.357)

90. Earl HM *et al.* 2019 6 versus 12 months of adjuvant trastuzumab for HER2-positive early breast cancer ( PERSEPHONE ): 4-year disease-free survival results of a randomised phase 3 non-inferiority trial. *Lancet* **393**, 2599–2612. (doi:10.1016/S0140-6736(19)30650-6)

91. Mavroudis D, Saloustros E, Malamos N, Kakolyris S, Boukovinas I, Papakotoulas P, Kentepozidis N, Ziras N, Georgoulias V. 2015 Six versus 12 months of adjuvant trastuzumab in combination with dose-dense chemotherapy for women with HER2-positive breast cancer : a multicenter randomized study by the Hellenic Oncology Research Group ( HORG ). *Ann. Oncol.* **26**, 1333–1340. (doi:10.1093/annonc/mdv213)

92. Rimawi M, Baylor Breast Care Center. 2016 NCT00999804 clinical trial registry results: Extension Study of Lapatinib Plus Herceptin With or Without Endocrine Therapy (HELEX). *ClinicalTrials.gov*. See https://clinicaltrials.gov/ct2/show/results/NCT00999804.

93. Vogel CL *et al.* 2002 Efficacy and Safety of Trastuzumab as a Single Agent in First-Line Treatment of HER2-Overexpressing Metastatic Breast Cancer. *J Clin Oncol* **20**, 719–726.

94. National Cancer Institute (NCI). 2015 NCT00513292 clinical trials registry results: Combination Chemotherapy and Paclitaxel Plus Trastuzumab in Treating Women With Palpable Breast Cancer That Can Be Removed by Surgery. *ClinicalTrials.gov*. See https://clinicaltrials.gov/ct2/show/results/NCT00513292.

95. Buzdar AU *et al.* 2013 Fluorouracil, epirubicin, and cyclophosphamide (FEC-75) followed by paclitaxel plus trastuzumab versus paclitaxel plus trastuzumab followed by FEC-75 plus trastuzumab as neoadjuvant treatment for patients with HER2-positive breast cancer (Z1041): A random. *Lancet Oncol.* **14**, 1317–1325. (doi:10.1016/S1470-2045(13)70502-3)

96. Minckwitz G Von *et al.* 2017 APHINITY trial (BIG 4-11): A randomized comparison of chemotherapy (C) plus trastuzumab (T) plus placebo (Pla) versus chemotherapy plus trastuzumab (T) plus pertuzumab (P) as adjuvant therapy in patients (pts) with HER2-positive early breast cancer (EBC). *J. Clin. Oncol.* **35**. (doi:10.1200/JCO.2017.35.18_suppl.LBA500)

97. Hoffmann-La-Roche. 2018 NCT01358877 clinical trials registry results: A Study of Pertuzumab in Addition to Chemotherapy and Trastuzumab as Adjuvant Therapy in Participants With Human Epidermal Growth Receptor 2 (HER2)-Positive Primary Breast Cancer (APHINITY). *ClinicalTrials.gov*.

98. Swain SM *et al.* 2015 Pertuzumab, Trastuzumab, and Docetaxel in HER2-Positive Metastatic Breast Cancer. *N. Engl. J. Med.* **372**, 724–734. (doi:10.1056/nejmoa1413513)

99. Genentech Inc. 2012 NCT00567190 clinical trials registry results: A Study to Evaluate Pertuzumab + Trastuzumab + Docetaxel vs. Placebo + Trastuzumab + Docetaxel in Previously Untreated HER2-positive Metastatic Breast Cancer (CLEOPATRA). *ClinicalTrials.gov*. See https://clinicaltrials.gov/ct2/show/results/NCT00567190.

100. Hurvitz SA *et al.* 2015 Combination of everolimus with trastuzumab plus paclitaxel as first-line treatment for patients with HER2-positive advanced breast cancer (BOLERO-1): A phase 3, randomised, double-blind, multicentre trial. *Lancet Oncol.* **16**, 816–829. (doi:10.1016/S1470-2045(15)00051-0)

101. Novartis (Novartis Pharmaceuticals). 2017 NCT01007942 clinical trials registry results: Daily Everolimus in Combination With Trastuzumab and Vinorelbine in HER2/Neu Positive Women With Locally Advanced or Metastatic Breast Cancer (BOLERO-3). *ClinicalTrials.gov*. See https://clinicaltrials.gov/ct2/show/results/NCT01007942.

102. National Cancer Institute (NCI). 2016 NCT00520975 clinical trial registry results: Bevacizumab in Treating Patients With Metastatic Breast Cancer That Overexpresses HER-2/NEU. *ClinicalTrials.gov*. See https://clinicaltrials.gov/ct2/show/results/NCT00520975.

103. Gianni L *et al.* 2013 AVEREL: A randomized phase III trial evaluating bevacizumab in combination with docetaxel and trastuzumab as first-line therapy for her2-positive locally recurrent/metastatic breast cancer. *J. Clin. Oncol.* **31**, 1719–1725. (doi:10.1200/JCO.2012.44.7912)

104. Hoffmann-La Roche. 2015 NCT00391092 clinical trials registry results: A Study of Avastin (Bevacizumab) in Combination With Herceptin (Trastuzumab)/Docetaxel in Patients With HER2 Positive Metastatic Breast Cancer. *ClinicalTrials.gov*. See https://clinicaltrials.gov/ct2/show/results/NCT00391092.

105. Fehrenbacher L *et al.* 2018 NSABP B-47 (NRG oncology): Phase III randomized trial comparing adjuvant chemotherapy with adriamycin (A) and cyclophosphamide (C) → weekly paclitaxel (WP), or docetaxel (T) and C with or without a year of trastuzumab (H) in women with node-positive or hi. *Cancer Res.* **78**, Abstract GS1-02. (doi:10.1158/1538-7445.SABCS17-GS1-02)

106. NSABP Foundation Inc. 2018 NCT01008150 clinical trials registry results: Phase II Randomized Trial Evaluating Neoadjuvant Therapy With Neratinib and/or Trastuzumab Followed by Postoperative Trastuzumab in Women With Locally Advanced HER2-positive Breast Cancer. *ClinicalTrials.gov*. See https://clinicaltrials.gov/ct2/show/results/NCT01008150.

107. Hoffmann-La Roche. 2016 NCT01026142 clinical trials registry results: A Study of a Combination of Trastuzumab and Capecitabine With or Without Pertuzumab in Patients With HER2-positive Metastatic Breast Cancer (PHEREXA). *ClinicalTrials.gov*. See https://clinicaltrials.gov/ct2/show/results/NCT01026142.

108. Rimawi M *et al.* 2018 First-Line Trastuzumab Plus an Aromatase Inhibitor, With or Without Pertuzumab, in Human Epidermal Growth Factor Receptor 2 – Positive and Hormone Receptor – Positive Metastatic or Locally Advanced Breast Cancer ( PERTAIN ): A Randomized , Open-Label Ph. *J. Clin. Oncol.* **36**, 2826–2835. (doi:10.1200/JCO.2017.76.7863)

109. Hoffmann-La Roche. 2017 NCT01491737 clinical trials registry results: A Study of Pertuzumab in Combination With Trastuzumab Plus an Aromatase Inhibitor in Participants With Metastatic Human Epidermal Growth Factor Receptor 2 (HER2)-Positive and Hormone Receptor-Positive Advanced. *ClinicalTrials.gov*. See https://clinicaltrials.gov/ct2/show/results/NCT01491737.

110. André F *et al.* 2014 Everolimus for women with trastuzumab-resistant, HER2-positive, advanced breast cancer (BOLERO-3): A randomised, double-blind, placebo-controlled phase 3 trial. *Lancet Oncol.* **15**, 580–591. (doi:10.1016/S1470-2045(14)70138-X)

111. Seagen Inc. 2020 NCT02614794 clinical trial registry results: A Study of Tucatinib vs. Placebo in Combination With Capecitabine & Trastuzumab in Patients With Advanced HER2+ Breast Cancer (HER2CLIMB). *ClinicalTrials.gov*. See https://clinicaltrials.gov/ct2/show/results/NCT02614794.

112. Shao Z *et al.* 2020 Efficacy, Safety, and Tolerability of Pertuzumab, Trastuzumab, and Docetaxel for Patients with Early or Locally Advanced ERBB2-Positive Breast Cancer in Asia: The PEONY Phase 3 Randomized Clinical Trial. *JAMA Oncol.* **6**, 3–8. (doi:10.1001/jamaoncol.2019.3692)

113. Harbeck N *et al.* 2016 Afatinib plus vinorelbine versus trastuzumab plus vinorelbine in patients with HER2-overexpressing metastatic breast cancer who had progressed on one previous trastuzumab treatment (LUX-Breast 1): An open-label, randomised, phase 3 trial. *Lancet Oncol.* **17**, 357–366. (doi:10.1016/S1470-2045(15)00540-9)

114. Boehringer Ingelheim. 2014 NCT01125566 clinical trials registry results: LUX-Breast 1: BIBW 2992 (Afatinib) in HER2-positive Metastatic Breast Cancer Patients After One Prior Herceptin Treatment. *ClinicalTrials.gov*. See https://clinicaltrials.gov/ct2/show/results/NCT01125566.

115. Rugo HS *et al.* 2021 Efficacy of Margetuximab vs Trastuzumab in Patients with Pretreated ERBB2-Positive Advanced Breast Cancer: A Phase 3 Randomized Clinical Trial. *JAMA Oncol.* **7**, 573–584. (doi:10.1001/jamaoncol.2020.7932)

116. Hoffmann-La Roche. 2016 NCT00545688 clinical trials registry results: A Study of Pertuzumab in Combination With Herceptin in Patients With HER2 Positive Breast Cancer. *ClinicalTrials.gov*. See https://clinicaltrials.gov/ct2/show/NCT00545688.

117. Puma Biotechnology I. 2017 NCT00915018 clinical trial registry: Study Evaluating Neratinib Plus Paclitaxel VS Trastuzumab Plus Paclitaxel In ErbB-2 Positive Advanced Breast Cancer (NEFERTT). *ClinicalTrials.gov*. See https://clinicaltrials.gov/ct2/show/NCT00915018.

118. Patel TA *et al.* 2019 A randomized, controlled phase II trial of neoadjuvant ado-trastuzumab emtansine, lapatinib, and nab-paclitaxel versus trastuzumab, pertuzumab, and paclitaxel in HER2-positive breast cancer (TEAL study). *Breast Cancer Res.* **21**, 1–9. (doi:10.1186/s13058-019-1186-0)

119. Masuda N, Toi M, Ueno T, Aogi K, Iwata H, Ohno S, Kuroi K, Sato N, Nakamura S. 2010 A multicenter, randomized phase II study of neoadjuvant chemotherapy including trastuzumab with cyclophosphamide with docetaxel in patients with operable HER2-positive breast cancer (JBCRG-10 study). *J. Clin. Oncol.* **28**. (doi:10.1200/jco.2010.28.15_suppl.tps105)

120. Sawaki1 M *et al.* 2011 Clinical Trial Notes Evaluation of Trastuzumab Without Chemotherapy as a Post-operative Adjuvant Therapy in HER2-positive Elderly Breast Cancer Patients: Randomized Controlled Trial [RESPECT (N-SAS BC07)]. *Jpn. J. Clin. Oncol.* **41**, 709–712. (doi:10.1093/jjco/hyr011)

121. Guarneri V *et al.* 2008 Multicentric, Randomized Phase III Trial of Two Different Adjuvant Chemotherapy Regimens plus Three Versus Twelve Months of Trastuzumab in Patients with HER2-Positive Breast Cancer (Short-HER Trial; NCT00629278). *Clin. Breast Cancer* **8**, 453–456. (doi:10.3816/CBC.2008.n.056)

122. Joensuu H *et al.* 2018 Effect of Adjuvant Trastuzumab for a Duration of 9 Weeks vs 1 Year With Concomitant Chemotherapy for Early Human Epidermal Growth Factor Receptor 2–Positive Breast Cancer The SOLD Randomized Clinical Trial. *JAMA Oncol.* **4**, 1199–1206. (doi:10.1001/jamaoncol.2018.1380)

123. Hoffmann-La Roche. 2014 NCT01419197 clinical trials registry results: A Study of Trastuzumab Emtansine in Comparison With Treatment of Physician’s Choice in Participants With HER2-positive Breast Cancer Who Have Received at Least Two Prior Regimens of HER2-directed Therapy (TH3R. *ClinicalTrials.gov*. See https://clinicaltrials.gov/ct2/show/results/NCT01419197.

124. Krop IE, Kim SB, González-Martín A, LoRusso PM, Ferrero JM, Smitt M, Yu R, Leung ACF, Wildiers H. 2014 Trastuzumab emtansine versus treatment of physician’s choice for pretreated HER2-positive advanced breast cancer (TH3RESA): A randomised, open-label, phase 3 trial. *Lancet Oncol.* **15**, 689–699. (doi:10.1016/S1470-2045(14)70178-0)

125. In press. HERTAX 2.

126. Amgen. 2019 NCT01901146 clinical trial registry results: Efficacy and Safety Study of ABP 980 Compared With Trastuzumab in Women With HER2-positive Early Breast Cancer (Lilac). *ClinicalTrials.gov*. See https://clinicaltrials.gov/ct2/show/NCT01901146.

127. Novartis Pharmaceuticals. 2019 NCT00968968 clinical trial registry results: Continued HER2 Suppression With Lapatinib Plus Trastuzumab Versus Trastuzumab Alone. *ClinicalTrials.gov*. See https://clinicaltrials.gov/ct2/show/results/NCT00968968.

128. Merck Sharp & Dohme Corp. 2018 NCT00963547 clinical trial registry results:A Study of MK-2206 in Combination With Trastuzumab and Lapatinib for the Treatment of HER2+ Solid Tumors (MK-2206-015). *ClinicalTrials.gov*. See https://clinicaltrials.gov/ct2/show/results/NCT00963547.

129. Novartis Pharmaceuticals. 2014 NCT00272987 clinical trial tregistry results: ErbB2 Over-expressing Metastatic Breast Cancer Study Using Paclitaxel, Trastuzumab, and Lapatinib. *ClinicalTrials.gov*. See https://clinicaltrials.gov/ct2/show/results/NCT00272987.

130. Daiichi Sankyo I. 2022 NCT03529110 clinical trial registry results: DS-8201a Versus T-DM1 for Human Epidermal Growth Factor Receptor 2 (HER2)-Positive, Unresectable and/or Metastatic Breast Cancer Previously Treated With Trastuzumab and Taxane [DESTINY-Breast03]. *ClinicalTrials.gov*. See https://clinicaltrials.gov/ct2/show/results/NCT03529110.

131. Hoffmann-La Roche. 2019 NCT02924883 clinical trial registry results: A Study to Evaluate the Efficacy and Safety of Trastuzumab Emtansine in Combination With Atezolizumab or Atezolizumab-Placebo in Participants With Human Epidermal Growth Factor-2 (HER2) Positive Locally Advance. *ClinicalTrials.gov*. See https://clinicaltrials.gov/ct2/show/NCT02924883.

132. Holmes FA *et al.* 2011 Correlation of molecular effects and pathologic complete response to preoperative lapatinib and trastuzumab, separately and combined prior to neoadjuvant breast cancer chemotherapy. *J. Clin. Oncol.* **29**, Abstract 506. (doi:10.1200/jco.2011.29.15_suppl.506)

133. Zhou Y, Li J, Peng S. 2016 Evaluation of tumor malignancy in patients with HER-2 overexpression breast cancer after TCH and TAC neoadjuvant chemotherapy. **22**, 123–127.

134. Hoffmann-La Roche. 2014 NCT01998906 clinical trial registry results: A Study of Herceptin (Trastuzumab) in Combination Chemotherapy in Women With Locally Advanced Breast Cancer. See https://clinicaltrials.gov/ct2/show/NCT01998906.

135. Untch M *et al.* 2010 Neoadjuvant treatment with trastuzumab in HER2-positive breast cancer: Results from the GeparQuattro study. *J. Clin. Oncol.* **28**, 2024–2031. (doi:10.1200/JCO.2009.23.8451)

136. Yamamoto Y *et al.* 2021 Abstract PD3-11: A randomized, open-label, phase III trial of pertuzumab re-treatment in HER2-positive, locally advanced/metastatic breast cancer patients previously treated with pertuzumab, trastuzumab, and chemotherapy: The Japan Breast Cancer Research. *Cancer Res.* **81**, PD3-11. (doi:https://doi.org/10.1158/1538-7445.SABCS20-PD3-11)
